# Assessment of sperm quality traits in relation to fertility in boar semen

**DOI:** 10.1186/1751-0147-51-53

**Published:** 2009-12-16

**Authors:** Neringa Sutkeviciene, Vita Riskeviciene, Aloyzas Januskauskas, Henrikas Zilinskas, Magnus Andersson

**Affiliations:** 1 Department of Non-infectious diseases, Faculty of Veterinary Medicine, Lithuanian Veterinary Academy, Kaunas, Lithuania; 2 Department of Production Animal Medicine, University of Helsinki, Saarentaus, Finland

## Abstract

**Background:**

Several studies have been published where sperm plasma membrane integrity correlated to fertility. In this study we describe a simple fluorometer-based assay where we monitored the fluorescence intensity of artificially membrane-ruptured spermatozoa with a fixed time staining with fluorescent DNA dyes.

**Methods:**

Membrane-impermeant fluorescent dyes Hoechst 33258 (H258) and propidium iodide (PI) were used to measure the fluorescence of the nucleus in artificially membrane ruptured spermatozoa and membrane-permeant dye Hoechst 33342 (H342) was used to measure fluorescence of intact spermatozoa. The concentration of spermatozoa in insemination doses varied from 31.2 × 10^6^/ml to 50 × 10^6^/ml and the average value was 35 × 10^6^/ml. Each boar was represented by three consecutive ejaculates, collected at weekly intervals. Nonreturn rate within 60 days of first insemination (NR %) and litter size (total number of piglets born) of multiparous farrowings were used as fertility measures.

**Results:**

Sperm fluorescence intensity of H258 and H342, but not the fluorescence intensity of PI-stained spermatozoa correlated significantly with the litter size of multiparous farrowings, values being r = - 0.68 (P < 0.01) for H258, r = - 0.69 (P < 0.01) for H342 and r = - 0.38, (P = 0.11) for PI.

**Conclusions:**

The increase in fluorescence values of membrane-ruptured H258 and unruptured H342-stained spermatozoa in boar AI doses can be associated with smaller litter size after AI. This finding indicates that the fluorescence properties of the sperm nucleus could be used to select for AI doses with greater fertilizing potential.

## Background

Assessing fertilizing potential of an ejaculate generally includes tests of sperm function, as well as evaluation of sperm morphology, motility profiles, concentration, viability, ability to acrosome-react and to penetrate oocytes [1]. Sperm morphology, sperm concentration and sperm motility are the three major components of routine sperm quality assessment. Microscopic assessment of sperm morphology, concentration and motility is inexpensive, however subjective and of low predictive power to monitor testicular function of boars and bulls as spermatozoa are not analyzed in terms of their total integrity [2-4].

Assessment of sperm plasma membrane integrity is one of the key parameters in evaluation of spermatozoal quality in relation to fertility in a particular male [5]. Plasma membrane is responsible for the preservation of cellular homeostasis; in this way the plasma membrane integrity exerts a vital role on sperm survival inside the female reproductive tract and on preservation of sperm fertilizing capacity [4,6]. One of the major features discriminating dead from live cells is loss in physical integrity of their plasma membranes and loss of motility [7].

The vast majority of methods used to assess cellular integrity are those based on the dye exclusion principle: some dyes such as PI and H258, are not able to pass plasma membrane of live cells, but enter dead cells and intercalate in their DNA, thus are used in assessing the structural integrity of the sperm plasma membrane [4,8]. The other - H342 is a relatively non-toxic membrane-permeant nucleic acid dye that is mainly used for cell-cycle studies, chromosome and fluorescent cytological analyses of DNA [9,10]. Many techniques employing above-mentioned and plethora of other fluorescent dyes are to be combined with microscopic or flow cytometric assessment techniques. Microscopic methods have disadvantages of subjectivity and speed of the analysis [11-13], while high running and purchase costs of a flow cytometer impede their application on a higher scale [14,15]. Still, techniques that are less time-consuming and of low running costs are of interest for routine application in AI station's work. Fluorometry is valuable alternative to flow cytometric and microscopic evaluation methods. Fluorometric methods of quantification of emitted fluorescence light of stained cells were shown to be accurate and robust enough to be applied to assess quality of semen quality used for AI [15,16].

The aim of the present study was to evaluate the relationship between fluorescence intensities of Hoechst and PI-stained spermatozoa in relation to litter size of multiparous farrowings.

## Materials and methods

### Experimental design

Semen from 19 boars, of which 8 were of Finnish Landrace and 11 of Yorkshire breed, housed at the same AI station, was used in this trial. The average age of the boars was 26.8 ± 8.5 months (range 13 to 50 months). Three semen samples were collected from each boar using gloved-hand technique, within the regular collecting schedule - once a week - at the boar station. Fresh ejaculated semen was diluted to approximately 35 × 10^6 ^spermatozoa/ml, with X-cell extender (IMV Technologies, L'Aigle Cedex, France) and placed in 90 ml plastic tubes. One AI dose from each ejaculate was transported to the laboratory and stored at 17°C in a Unitron climate box (Unitron Skandinavia S/A) in closed plastic tubes until examination. The rest - were used for AI in commercial farms where sows were inseminated with fresh semen generally twice per oestrus 15 - 24 hours apart. Analysis of sperm motility, morphology, and plasma membrane integrity and fluorescence intensity was conducted at 24 h following semen collection and processing.

### Fertility data

Fertility data were obtained from the Agricultural Data-Processing Center Ltd. (Vantaa, Finland). Each of 19 boars was used for at least 50 first inseminations in recorded herds and had ≥ 12 litters of recorded farrowings. In total, 2296 first inseminations and 1114 litters were recorded from all inseminations in approximately 110 commercial farms. Nonreturn rate within 60 days of first insemination (NR %) and litter size (total number of piglets born) of multiparous farrowings were used as fertility measures.

### Semen analysis

#### Sperm morphology

At the semen laboratory the sperm morphology was evaluated in air-dried Giemsa stained smears according to Watson [17]. In total, 200 spermatozoa were examined. All abnormalities on any given spermatozoon were counted and then were divided into four groups according Blom [18], that is: normal spermatozoa, spermatozoa with major sperm defects (abnormalities of sperm head and acrosome, coiled sperm tail, etc.), spermatozoa with proximal droplets, and spermatozoa with minor sperm defects (simple bent tail, loose sperm head, etc.). Morphological abnormalities were expressed as a percentage of the total number of all counted spermatozoa.

#### Sperm motility

Sperm motility was evaluated both subjectively and using a computer-assisted semen analyzer (CASA) (Sperm Vision Minitube™ of America, Inc., 2002). For the analysis, a 300-μl aliquot of the thoroughly but gently mixed semen sample was placed into an open 3-ml tube. The tube was kept in a 35°C water bath (Grants Instruments Ltd., Cambridge, UK) for 5 min before semen analyses. A 5-μl aliquot was placed on a pre-warmed 38°C microscope slide, covered with a coverslip (24 × 24 × 1.5 mm) and the proportions of total motile spermatozoa were recorded.

#### Fluorescent dyes

Calcein AM (CAM), propidium iodide (PI), Hoechst 33258 (H258) and Hoechst 33342 (H342) dyes were purchased from Molecular Probes Inc. (Eugene, OR, USA). One milligram of CAM was diluted in 1 ml of dimethyl sulfoxide (DMSO) (Mallincrodt Bacer B.V.), mixed for 10 min, kept in the dark, and then stored in 10-μl aliquots at -20°C. Twenty milligrams of PI were diluted in 1 l BTS (Beltsville Thawing Solution, Kubus S.A., Spain) and stored in 3-ml aliquots at -20°C. Six milligrams of H258 were diluted in 200 ml of BTS, mixed for 30 min in the dark, and stored in 2-ml aliquots at -20°C. Six milligrams of H342 were diluted in 200 ml of BTS, mixed for 30 min in the dark, and stored in 2-ml aliquots at -20°C. Before use, the dyes were thawed in a dark chamber at 35°C (Thermax, B8000, Bergen, Norway).

#### Assessment of plasma membrane integrity

Microscopic evaluation of plasma membrane integrity was carried out with a combination of two fluorescent stains, CAM and PI, according to Januskauskas and Rodriguez-Martinez [19], but using PI instead of Ethidium homodimer-1. Briefly, 10 μl of CAM (1 mg/ml) were mixed with 500 μl of BTS and added to 500 μl of PI (0.02 mg/ml) in BTS. For staining, 100-μl aliquots of semen were placed in 3-ml tubes, and 100 μl of CAM/PI solution was added. Each sample was further incubated for 10 min in the dark at 35°C. Sub-samples of 5 μl of the stained suspension were placed on clean microscope slides and overlaid carefully with coverslips. The slides were then evaluated under an epifluorescence microscope (Olympus BH2 with epifluorescence optics, Olympus Optical Co., Ltd., Japan) using ×500 magnification. In each slide 200 spermatozoa were categorized to CAM-stained green (live) and PI-stained red (dead) and the percentage of viable spermatozoa was then calculated.

#### Assessment of the fluorescence of the sperm nucleus

H258 and PI were used to measure the fluorescence intensity of the sperm nuclei in artificially membrane-ruptured spermatozoa. In contrast, membrane-permeant H342 was used to measure fluorescence intensities of intact spermatozoa. Fluorescence outputs were recorded in a fluorometer (Fluoroscan Ascent, Thermo Labsystems Oy, Vantaa, Finland) at 32°C. In order to estimate fluorescence intensities of given semen samples, sperm membranes ought to be disrupted. For this, 500-μl aliquots of semen were placed in 3-ml tubes and subjected to rapid freezing and slow thawing which induced membrane damage. The tubes were rapidly frozen by immersion directly into liquid nitrogen for 1 min. Thereafter the tubes were kept at room temperature for 30 sec, before being placed in a 35°C water bath for 3 min., as described previously by Alm et al. [15].

For the analysis, 50-μl aliquots of the artificially membrane-ruptured spermatozoa, plus 50 μl of PI or H258 were dispensed into the wells of a microtiter plate (Black Microtiter Plate 96 wells, Thermo Labsystems Oy, Vantaa, Finland) in three replicates. Blanks containing 50 μl of X-cell extender (IMV Technologies, France) and 50 μl of PI or H258 solution were dispensed in four replicates. The plate was then gently shaken for 2 min and further incubated in the fluorometer for 8 min at 32°C. Eleven samples and their corresponding blanks were analyzed at each assessment session. Semen samples of each boar were also stained with H342 in the same manner as with H258 except that membranes were not disrupted. The interference filter at the excitation path and the emission filter had maximum transmissions at 544 nm and 590 nm for PI, and 355 nm and 460 nm for H258 and H342. Sperm concentration in each AI dose was confirmed in a Bürker haemocytometer chamber (Fortuna, Germany). The results were expressed as fluorescence value/million spermatozoa.

#### Fluorometer-based assessment of membrane integrity

The fluorescence - based viability was assessed according to Alm et al. [15]. Briefly, fluorescence intensities of PI and H258 were recorded in artificially killed and live semen samples. Percentage of viable spermatozoa was calculated based on the ratio of fluorescence output of intact and of killed subsamples, corrected in relation to background fluorescence (blank) [20].

### Statistical analysis

Statistical analyses were carried out using SPSS software (version 13.0 for Windows, SPSS Inc., Chicago, IL, USA). Descriptive statistics, two-sample analysis, and Spearman rank correlations were calculated. The Spearman rank correlations were used to calculate the relationships between the sperm quality traits and fertility. Relationship between sperm viability and fertility results was represented by scatter diagram. Values are presented as means ± standard deviation (SD) and were considered statistically significant when P < 0.05.

## Results

The average sperm viability of microscopically evaluated CAM and PI stained semen was 90.6 ± 2.3%. The average fluorometer-assessed PI and H258 sperm viability was 89.0 ± 3.7% for PI-stained semen and 86.4 ± 5.2% for H258 stained samples.

**Figure 1:**
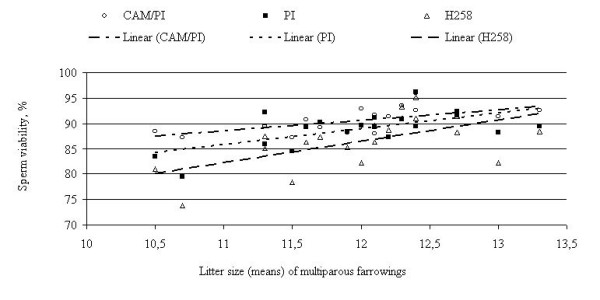
**Relationship between sperm viability assessment methods and litter size of multiparous farrowings**. Circle indicates microscopically assessed sperm viability results based on CAM/PI staining; Square indicates fluorometrically-assessed sperm viability results based on PI staining; Triangle indicates fluorometrically-assessed sperm viability results based on H258 staining. The lines show the trend in the data.

The relationship between the percentage of viable spermatozoa and litter size of multiparous sows is shown in Fig. 1. Sperm viability of microscopically - assessed CAM and PI stained semen correlated significantly (r = 0.68, P < 0.05) with litter size, but not with nonreturn rate (NR%). Fluorometric assessment of sperm viability correlated significantly r = 0.51, (P < 0.05) and r = 0.63, (P < 0.05) with litter size for PI and H258 staining respectively. The results from CAM/PI, PI and H258 staining were highly intercorrelated. There was a significant correlation between litter size and total CASA-assessed sperm motility r = 0.59, (P < 0.05).

The basic sperm quality parameters did not differ significantly (P > 0.05) between ejaculates of each individual boar (data not shown). Mean value of nonreturn rate within 60 days of first insemination was 82.2 ± 3.8% (Min 77.0% - Max 89.0%). A summary of basic semen quality and fertility parameters is shown in Table 1.

**Table 1:** Summary of sperm parameters in AI dose and field fertility, data presented as mean ± SD (n = 19 boars).

	Mean ± SD	Min-Max
Sperm concentration (million/ml)	36.9 ± 1.8	33.9-39.8
Major sperm defects (%)	3.0 ± 2.6	0.5-11.0
Minor sperm defects (%)	11.2 ± 8.7	2.0-33.7
Morphologically normal spermatozoa (%)	84.8 ± 10.3	55.0-96.0
Proximal droplets (%)	1.1 ± 0.8	0.2-27
Subjective motility (%)	77.1 ± 4.6	66.7-85.3
CASA-assessed total motility (%)	91.2 ± 5.9	75.4-95.8
Litter size of multiparous farrowings	12.0 ± 0.7	10.5-13.3
Nonreturn rate within 60 days of first insemination (%)	82.2 ± 3.8	77.0-89.0

The mean fluorescence intensity of fluorometrically-assessed membrane ruptured spermatozoa was 0.6 ± 0.1 relative fluorescence units/10^6 ^spermatozoa for PI, 21.9 ± 1.9 relative fluorescence units/10^6 ^sperm for H258-stained samples respectively. Mean fluorescence intensities of H342 stained membrane un-ruptured spermatozoa was 22.1 ± 3.6 relative fluorescence units/10^6 ^spermatozoa.

The fluorescence intensity of H258 - stained membrane ruptured spermatozoa and the fluorescence intensity of H342 - stained un-ruptured spermatozoa correlated inversely with litter size (Table. 2). Fluorescence of PI-stained spermatozoa did not correlate significantly with litter size (r = - 0.38, P = 0.11). There was no significant correlation between fluorescence intensities of artificially membrane-ruptured and un-ruptured spermatozoa and NR %.

**Table 2:** Correlation coefficients and levels of significance between different methods of staining used to evaluate the fluorescence intensity boar spermatozoa and field fertility after AI (n = 19 boars).

	PI	H258	H342	LS
PI	-	0.69**	0.18	-0.38
H258	0.69**	-	0.58**	-0.68**
H342	0.18	0.58**	-	-0.69**

* = P < 0.05; ** = P < 0.01

PI = the fluorescence intensity of propidium iodide - stained membrane ruptured spermatozoa derived from fluorometric assessment.

H258 = the fluorescence intensity of H258 - stained membrane ruptured spermatozoa derived from fluorometric assessment.

H342 = the fluorescence intensity of H342 - stained un-ruptured spermatozoa derived from fluorometric assessment.

LS = litter size of multiparous farrowings.

There was a significant boar effect on sperm assessment values of PI, H258 and H342 - stained spermatozoa (P < 0.05) and for CASA results (P < 0.05).

## Discussion

Assessment of sperm function in a semen sample has an ultimate goal: to disclose its potential fertility and, in the long run, to disclose the fertility of the male from whom the sample has been collected [21]. The relationship between laboratory semen characteristics and fertility has been extensively discussed and reviewed [3,22-25]. Classical methods of semen evaluation have low power in predicting sperm fertility, because only the samples with markedly inferior quality can be detected [26]. Even if one method of semen evaluation has precise and accurate data that give a high correlation between one or several laboratory tests and fertility, the test or tests still might not be useful for predicting fertility [27]. For this reason, methods for evaluating semen quality before a boar semen is collected, or prior to distribution of his semen for insemination, are undergoing continuous development in an effort to estimate this "fertility potential" [4].

In our study a new application of a fluorometer-based method for measuring the fluorescence of the sperm nucleus is described. Most frequently used dead cell dye PI gave no significant correlation between semen sample fluorescence and litter size. Other DNA specific fluorescent probes as H258 and H342 may also be used to determine plasma membrane integrity. Our results showed that both the fluorescence output of un-ruptured spermatozoa stained with H342 and the fluorescence output of artificially ruptured spermatozoa stained with the H258 gave similar significant correlations with litter size. Both the H258 and H342 are minor groove-binding DNA stains [10,13,28,29]. Pintado et al. [5] showed that PI stained more sperm cells than H258.

The novelty in our study is that we could demonstrate that fluorescence output of AI doses could be useful parameter to select for higher litter-size with either of the used Hoechst stains. The higher fluorescence of spermatozoa in boars with smaller litter size might be explained by a deficiency in the process of chromatin condensation in a larger number of spermatozoa of a gived semen AI dose. Normal structure of sperm chromatin is essential for the fertilizing ability of spermatozoa *in vivo *[30]. Sperm chromatin structural integrity of several animal species and a man [12,31-33] has been extensively studied and has been shown to be correlated with fertility.

Sperm motility parameters are very important semen characteristics. Correlations between the results of various laboratory assays therein motility to the male fertility have been reported since 1950s [34]. The possibility of computerized measurement of spermatozoa motility by computer-assisted semen analysis CASA enable to measure motility characteristics of individual spermatozoa [35]. CASA provide an objective and useful information about sperm function [9]. In our study the total sperm motility assessed with the CASA correlated significantly with litter-size, although in a slightly lower degree than did the fluorescence intensity of Hoechst-stained spermatozoa. It has been previously demonstrated that sperm motion characteristics, obtained by CASA, have been correlated with the sperm penetration of human oocytes and the results of *in vitro *fertilization [36]. Tardif et al. [1] have shown that sperm motility (the percentage of motile spermatozoa assessed visually by microscopy) prior to thermal stress was well-correlated to fertility rates. Jasko et al. [37] performed the most comprehensive study of the relationship between conventional semen quality parameters and fertility and, they found reasonable correlations between the percentages of motile (r = 0.40), progressively motile (r = 0.46) and morphologically normal (r = 0.36) sperm with fertility results.

The comparison of the three methods used to assess the plasma membrane integrity revealed that the microscopic evaluation of plasma membrane integrity carried out with a combination of two fluorescent dyes, CAM and PI had the highest significant correlation coefficient with litter-size, but not with NR %. Similar was observed by Berger et al. [38] who also observed no relationship between the percentage of live spermatozoa, assessed using H258, and *in vivo *fertility, from heterospermic inseminations. Our results from CAM/PI, PI and H258 staining were highly intercorrelated.

In our studies differences among the individual boars on sperm viability derived from fluorometric assessment by using propidium iodide and H258; on the fluorescence intensity of H258 and of H342-stained spermatozoa and sperm motility parameters, were found. Differences among the individual boars in the proportions of sperm staining with R123/propidium iodide and H258, representing the living and dead sperm populations were found by Fraser et al. [29].

The ultimate goal of *in vitro *semen quality assessment is to predict fertility outcome. Still, the present approach is to combine several semen quality tests to have complex information of AI semen samples. Another task is to standardize different semen *in vitro *assessment tests, so that different laboratories could get the comparable results. Our results show that fluorometric assessment of Hoechst-stained spermatozoa may be an optimal approach. Tartaglione and Ritta [39] suggest that the higher the number of analyses performed, the better the prediction of fertility capability.

The main finding of this paper is that the fluorescence value/million spermatozoa of H258 and H342 stained spermatozoa assessed by an automatic fluorometer correlates with litter-size of multiparous farrowings. The present sperm evaluation test is less labor intensive and less subjective compared to conventional microscopic semen quality analysis.

## Competing interests

The authors declare that they have no competing interests.

## Authors' contributions

NS carried out the study, compiled the results and drafted the manuscript. VR participated in statistical analysis of the data and has helped to draft the manuscript. AJ and HZ were involved significantly in the study, interpreting data and composing the manuscript. MA coordinated the study. He has been involved in many of the studies reviewed in this manuscript, and also helped to draft the manuscript. All authors read and approved the final manuscript.
